# MiR-200b Suppresses Gastric Cancer Cell Migration and Invasion by Inhibiting NRG1 through ERBB2/ERBB3 Signaling

**DOI:** 10.1155/2021/4470778

**Published:** 2021-09-07

**Authors:** Tonglei Xu, Fangliang Xie, Dazhou Xu, Weidong Xu, Xuming Ge, Shengxiang Lv, Shouying Li

**Affiliations:** ^1^Department of Hepatobiliary Surgery, First People's Hospital of Lianyungang, Lianyungang 222061, Jiangsu Province, China; ^2^Department of Gastroenterology, First People's Hospital of Lianyungang, Lianyungang 222061, Jiangsu Province, China

## Abstract

**Purpose:**

Accumulating evidence indicates that miRNAs (miRs) play crucial roles in the modulation of tumors development. However, the accurately mechanisms have not been entirely clarified. In this study, we aimed to explore the role of miR-200b in the development of gastric cancer (GC).

**Methods:**

Western blot and RT-PCR were applied to detect epithelial-mesenchymal transition (EMT) marker expression and mRNA expression. Transwell assay was used for measuring the metastasis and invasiveness of GC cells. TargetScan system, luciferase reporter assay, and rescue experiments were applied for validating the direct target of miR-200b.

**Results:**

MiR-200b was prominently decreased in GC tissues and cells, and its downregulation was an indicator of poor prognosis of GC patients. Reexpression of miR-200b suppressed EMT along with GC cell migration and invasion. Neuregulin 1 (NRG1) was validated as the target of miR-200b, and it rescued miR-200b inhibitory effect on GC progression. In GC tissues, the correlation of miR-200b with NRG1 was inverse.

**Conclusion:**

MiR-200b suppressed EMT-related migration and invasion of GC through the ERBB2/ERBB3 signaling pathway via targeting NRG1.

## 1. Introduction

As a malignant tumor originating from the gastric mucosa epithelium, gastric cancer (GC) occupies the first place among various malignant tumors in China [1, 2]. The vast majority of GC belongs to adenocarcinoma, and there are no obvious symptoms at the early stage. It is often similar to gastritis, gastric ulcer, and other chronic gastric disease symptoms, which are easy to be ignored. Therefore, the early diagnosis rate of GC is still low in China [3, 4]. Although significant improvements including surgery, radiotherapy, and targeted therapy have been made in treating GC, most patients will experience postoperative recurrence and metastasis, leading to poor survival [5]. Thus, it is imperative to investigate the metastatic mechanism in GC.

MiRNAs (miRs) have been proved to serve as oncogenes or tumor suppressors via modulating the expression of target genes at the posttranscriptional level in various tumors [6, 7]. Accumulating evidence has shown that miRs take part in many tumors development, including migration and invasion. For example, Ye et al. displayed that downregulation of miR-7 facilitated GC metastasis via promoting p65-mediated NF-*κ*B activation [8]. Also, miR-214 was decreased in GC, and its downregulation contributed to GC cell migration and invasion via inducing EMT [9]. Moreover, miR-1292 suppressed GC cell invasion and migration via DEK [10]. Furthermore, miR-200b was reported to take part in GC development [11]. However, the molecular mechanism of miR-200b in the modulation of GC invasion and migration has not been fully clarified. Epithelial-mesenchymal transition (EMT) plays important roles in the metastasis of various cancers, and it is an important biological process in which epidermal malignant cells acquire the ability to migrate and invade [12–14]. Thus, understanding the mechanism of miR-200b in regulating EMT is very important.

Neuregulin 1(NRG1), a member of the NRG family, is an emerging potential oncogene in tumors [15]. NRG1 has been proved to participate in the progression of multiple cancers [16, 17]. For instance, Jones et al. displayed that NRG1 played important roles in the treatment for metastatic cancer [16]. Moreover, Yun et al. showed that NRG1 served as a potential biomarker for prognosis and treatment of GC patients [18]. However, the role of NRG1 in GC progression has not been reported. NRG1 was verified as the target of miR-125 in the regulation of the apoptosis and invasion of glioma [19]. Besides, miR-296 targeted NRG1 to suppress hepatocellular carcinoma progression [20]. Here, we investigated the role of NRG1 and its underlying mechanism in the modulation of GC cell migration and invasion.

Previous studies have been reported that NRG1 played important roles in cancers by directly binding to ERBB3 or ERBB4 and ERBB3 or ERBB4 interacts with ERBB2 or ligand-receptor, leading to receptor phosphorylation and signal cascade activation [21]. In the present study, we explored miR-200b's role in GC progression and explored whether the ERBB2/ERBB3 signaling pathway was involved in GC progression modulated by the miR-200b/NRG1 axis.

## 2. Methods

### 2.1. Study Design

From January 2014 to December 2014, 60 patients with GC resection were recruited in the First People's Hospital of Lianyungang. No patients had received preoperative chemotherapy or targeted therapy. Before collection of tissue specimens, written informed consent should be signed by all patients, and the ethics committee of the First People's Hospital of Lianyungang approved this study. The GC specimens were determined by pathologists, and they were stored at −80°C for further analysis.

### 2.2. Cell Culture and Cell Transfection

Two GC cell lines (MGC-803 and BGC-823) and normal gastric epithelial cell (GES-1) were provided by the Cell Bank of the Chinese Academy of Science (Shanghai, China). All cells were maintained in the RPMI-1640 medium supplemented with 10% FBS (Fatal bovine serum) at 37°C and 5% CO_2_.

MiR-200b mimic/inhibitor or NRG1 siRNA/vector synthesized by RiboBio (Guangzhou, China) was transfected into MGC-803 and BGC-823 cells, respectively. The transfection was conducted for 48 h with the aid of Lipofectamine 2000 (Invitrogen, Carlsbad, USA) following the manufacturer's instructions.

### 2.3. Real-Time PCR (RT-PCR) Assay

Total RNA was isolated by Trizol reagent (Invitrogen, Grand Island, USA). The synthesis of cDNA was performed using a M-MLV Reverse Transcriptase Kit (Invitrogen). RT-PCR was performed using an SYBR Green Real-Time PCR Assay Kit (ThermoFisher, Waltham, USA). The sequences of primers were as follows: miR-200b-F: 5′-CACACTGAAATCCTGTCAGCTTC-3′, miR-200b-R: 5′- ACGUGACACGUUCGGAGAATT-3′; NRG1-F: 5'-CGGTGTCCATGCCTTC CAT-3', NRG1-R: 5'-GTGTCA CGAGAAGTAGAGGTCT-3'; U6-F: 5′-GCTT CGGCAGCACATATACTAAAAT-3′, U6-R: 5′-CGCTTCACGAATTTGCGTG TCAT-3′; and GAPDH-F: 5'-GGGGCTCTCCA GAACATCATCC-3', GAPDH-R: 5'-ACGCCTGCTTCACCACCTCTT-3'. U6 and GAPDH were served as the internal control. Relative expression was calculated using the 2^−ΔΔCt^ method.

### 2.4. Western Blotting Assay

Total protein was extracted from cells or tissue specimens. After the proteins were separated by SDS-PAGE, followed by transfer to NC membranes, they were blocked with 5% skimmed milk for 1 h. Subsequently, the membranes were incubated with primary antibodies at 4°C overnight, followed by second antibodies for 1 h. Lastly, BeyoECL (Thermo Fisher Scientific) was applied for detecting the immune complexes. GAPDH assay served as internal control.

### 2.5. Transwell Assay

Transwell migration assay and Transwell invasion assay were almost the same except for the membranes with or without 25 *µ*g Matrigel coating. A total of 5 × 10^4^ cells were placed onto the upper chamber, and the complete medium containing with 5% FBS was seeded in the lower chamber as a chemoattractant. After incubation for 24 h, the cells in the upper chamber migrated or invaded to the lower chamber. The cells in the lower chamber were stained with crystal violet, photographed, and calculated under a light microscope.

### 2.6. Dual-Luciferase Reporter Assay

Firstly, we amplified the 3'-UTR of NRG1 and then cloned it into a pGL3-reporter vector (Promega, WI, USA), named NRG1-WT. The 3'-UTR mutant type of NRG1 (NRG1-MUT) was generated by using the site-directed mutagenesis kit (TaKaRa, Shiga, Japan). The HEK-293T cells were cotransfected with miR-200b mimic or inhibitor and NRG1-WT or -MUT by Lipofectamine 2000 (Invitrogen). After transfection for 48 h, the luciferase activity was tested by using the dual-luciferase reporter assay system (Promega, Madison, WI, USA). Renilla luciferase activity served as a reference control.

### 2.7. Statistics

Data were presented as mean ± SD. The values were analyzed by SPSS 22.0 software (IBM, NY, USA). MiR-200b high expression and low expression were cut by using the mean value. The chi square test or two-tailed Pearson's correlation analysis were applied to detect the correlation of miR-200b with the clinicopathological features of GC patients or miR-200b with NRG1, E-cadherin, or vimentin. Overall survival rate was analyzed by the Kaplan–Meier method, and the difference analysis between the survival curves was measured by the log-rank test. The statistical significance of differences between two groups was determined by Student's *t*-test, and when more than two groups, one-way analysis of variance (ANOVA) with Tukey's *post hoc* test was applied to determine the differences. *p* < 0.05 was considered as statistically significant.

## 3. Results

### 3.1. MiR-200b Was Negatively Correlated with GC Aggressiveness

**Figure 1:**
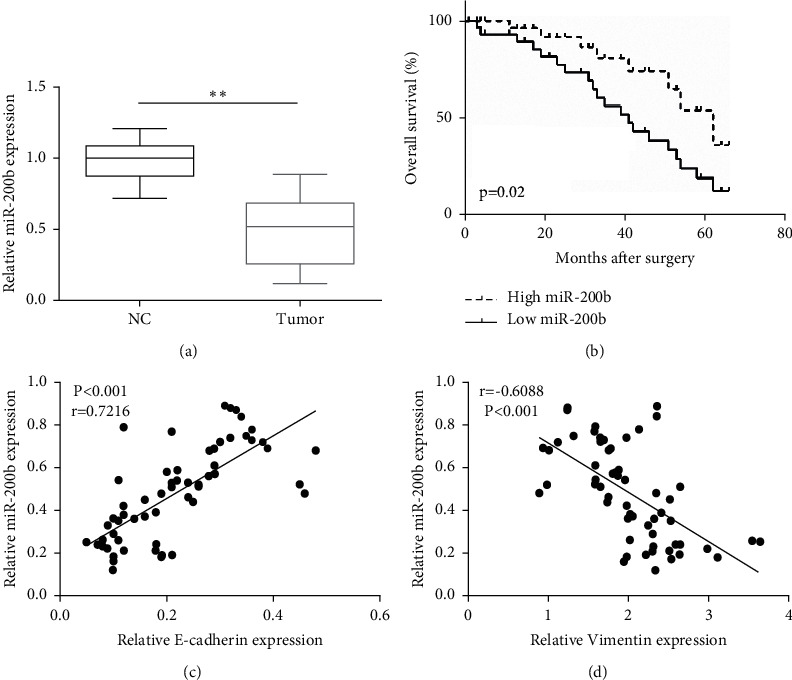
MiR-200b was negatively associated with GC aggressiveness. (a) MiR-200b expression was measured in GC tissue specimens and normal control (NC) tissue specimens (*n* = 60). (b) Overall survival was detected in GC patients with high or low miR-200b expression. (c) Positive association of miR-200b expression with EMT-related marker (E-cadherin) expression. (d) Negative association of miR-200b expression with EMT-related marker (vimentin) expression (^*∗∗*^*p* < 0.01).

To investigate whether miR-200b was abnormally expressed during GC metastasis, we first detected miR-200b expression in GC tissue specimens. As Figure 1(a) shows, miR-200b was dramatically decreased in GC tissues relative to the normal control group. The median value of miR-200b expression was used as the cutoff point to divide miR-200b expression into high and low expression of miR-200b. Then, we measured the clinical significance of miR-200b in GC. As Table 1 shows, miR-200b was significantly associated with clinical features of GC patients. Moreover, we revealed that high expression of miR-200b predicted high overall survival time, while low expression of miR-200b served as an indicator of poor prognosis of GC patients (Figure 1(b)). More strikingly, the relationship between miR-200b and E-cadherin was positive (Figure 1(c)), while miR-200b negatively correlated with vimentin expression (Figure 1(d)). These findings indicated that miR-200b might be a favorable diagnostic maker.

### 3.2. MiR-200b Inhibited GC Metastasis through EMT

**Figure 2:**
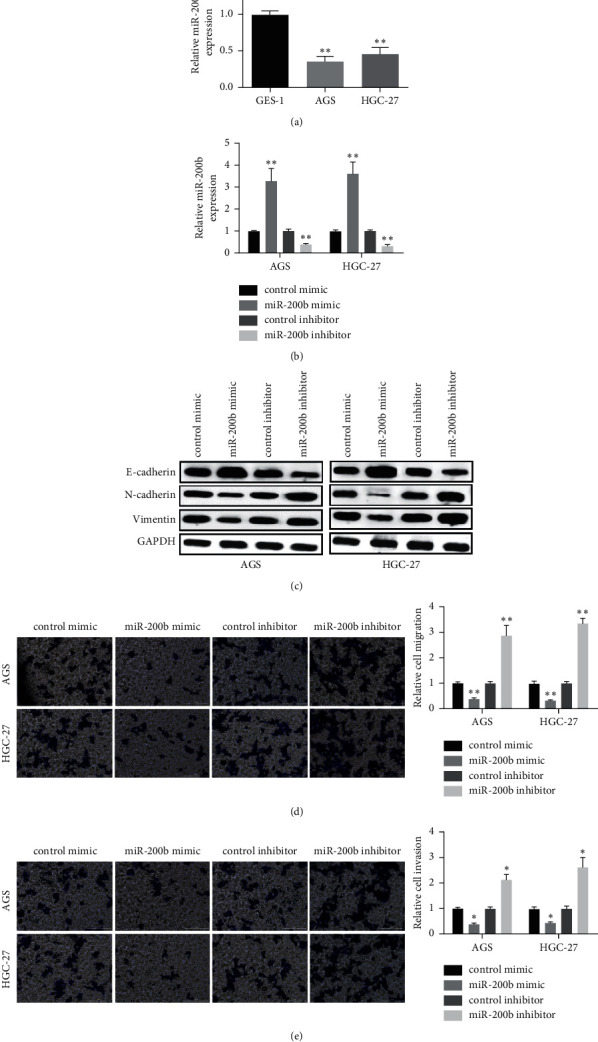
MiR-200b inhibited the invasion and migration of GC through EMT. (a) Relative expression of miR-200b tested in GC cells. (b) MiR-200b expression measured in GC cells treated with miR-200b mimic or inhibitor. (c) The protein levels of EMT markers tested in GC cells after treatment with miR-200b mimic or inhibitor. (d) GC cell migration and (e) invasion detected after transfected with miR-200b mimic or inhibitor (^*∗*^*p* < 0.05, ^*∗∗*^*p* < 0.01).

To verify the specific role of miR-200b in GC metastasis *in vitro*, miR-200b expressional level in GC cells (MGC-803 and BGC-823) was tested firstly. As Figure 2(a) shows, miR-200b was significantly downregulated in both two GC cell lines compared with normal cells (GES-1). Then, the two GC cells were transfected with miR-200b mimic or inhibitor to overexpress or knockdown of miR-200b expression. Results showed that the transfection was very successful (Figure 2(b)). EMT-related markers were then detected by western blot, and the results displayed that N-cadherin and vimentin level were reduced and E-cadherin level was elevated in miR-200b mimic GC cells, while N-cadherin and vimentin level were elevated and E-cadherin was reduced in miR-200b inhibitor GC cells (Figure 2(c)). Transwell assay was then applied for measuring the migration and invasion of GC cells. The findings displayed that miR-200b mimic decreased and miR-200b inhibitor increased the migratory ability of GC cells (Figure 2(d)). Similar results were observed in the invasion of GC cells after transfection with miR-200b mimic or inhibitor (Figure 2(e)). Correlatively, the observations implied that miR-200b showed a suppressive effect on the invasion and migration of GC through EMT.

### 3.3. NRG1 Was the Target of miR-200b

**Figure 3:**
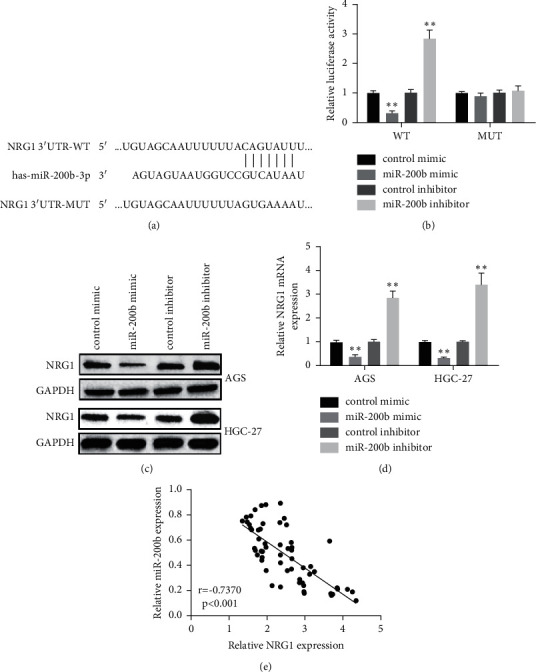
MiR-200b targeted NRG1. (a) Display of the NRG1 3'UTR-WT or -MUT sequences with miR-200b sequences. (b) Luciferase activity of NRG1 3'UTR-WT or -MUT in HEK-293T cells after increasing or decreasing miR-200b. (c) Protein level of NRG1 and (d) mRNA expression of NRG1 detected in GC cells after increasing or decreasing miR-200b. (e) Relationship between miR-200b expression and NRG1 expression (^*∗*^*p* < 0.05, ^*∗∗*^*p* < 0.01).

To explore the underlying mechanism of miR-200b in GC metastasis, bioinformatics approaches (miRanda and TargetScan) were applied for seeking for the target of miR-200b. As Figure 3(a) shows, NRG1 was the selected candidate target of miR-200b. Then, dual-luciferase reporter assay was selected to further verify whether NRG1 was the direct target of miR-200b. Results displayed that miR-200b mimic showed a decreased luciferase activity, while miR-200b inhibitor showed an increased luciferase activity in wild-type reporter, but not with mutant (Figure 3(b)), indicating that miR-200b modulated NRG1 by binding its 3'UTR. Furthermore, NRG1 protein (Figure 3(c)) and mRNA level (Figure 3(d)) were reduced by miR-200b mimic, while elevated by miR-200b inhibitor in both two GC cell lines. Moreover, the findings displayed that miR-200b and NRG1 were negatively correlated in GC tissues (Figure 3(e)). These observations suggested that NRG1 was the direct target of miR-200b.

### 3.4. NRG1 Rescued the MiR-200b Effect on GC EMT and Metastasis

**Figure 4:**
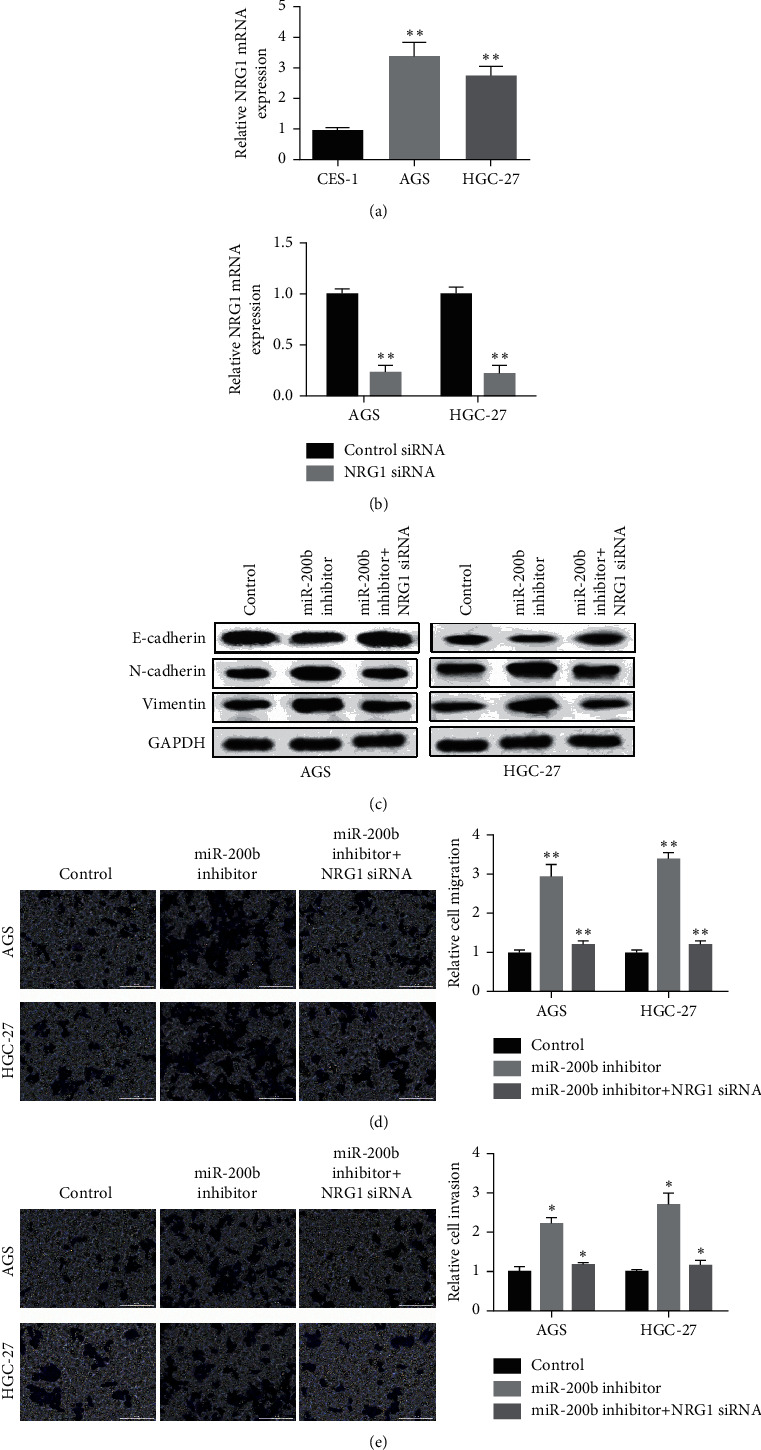
NRG1 overturned the miR-200b effect on GC cell invasion and migration. (a) Relative expression of NRG1 tested in two GC cells. (b) Detection of NRG1 expression in GC cells after silence NRG1. (c) The protein levels of EMT markers detected in GC cells after transfected with miR-200b inhibitor or combined with NRG1 siRNA. (d) Cell migration and (e) cell invasion detected in GC cells after transfected with miR-200b inhibitor or combined with NRG1 siRNA (^*∗*^*p* < 0.05, ^*∗∗*^*p* < 0.01).

To uncover the role of NRG1 in the EMT and metastasis modulated by miR-200b *in vitro*, NRG1 expressional level in GC cells was measured firstly. As shown in Figure 4(a), NRG1 was upregulated obviously in both two GC cell lines compared with normal cells. Then, GC cells were transfected with NRG1 siRNA to silence NRG1 expression. As Figure 4(b) shows, the transfection was very successful. EMT-related markers were then detected by western blot, and the results displayed that N-cadherin and vimentin level was elevated and E-cadherin level was reduced in miR-200b inhibitor GC cells, while their expression was reversed by NRG1 siRNA (Figure 4(c)). Transwell assay was then applied to measure the migration and invasion of GC. The findings displayed that miR-200b inhibitor increased, while combined with NRG1 siRNA decreased the migratory ability of GC cells (Figure 4(d)). Similar results were found in cell invasion in GC cells after transfection with miR-200b inhibitor or combining with NRG1 siRNA (Figure 4(e)). Correlatively, these observations implied that NRG1 overturned the miR-200b effect on GC cell invasion and migration through EMT.

### 3.5. ERBB2/ERBB3 Pathway Was Critical for MiR-200b Biological Behavior in GC

**Figure 5:**
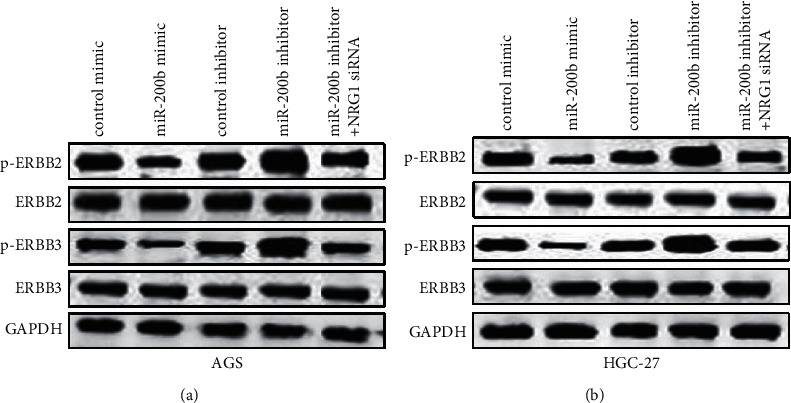
MiR-200b modulated GC cell migration and invasion through the ERBB2/ERBB3 signaling pathway. (a), (b) The protein level of p-ERBB2, ERBB2, p-ERBB3, and ERBB2 measured in GC cells after increasing miR-200b, decreasing miR-200b, or combined with decreasing NRG1.

As described above, NRG1 and ERBB2 or ERBB3 interaction could activate a series of signaling, resulting in cell migration and invasion [22]. Here, we investigated whether the NRG1/ERBB2/ERBB3 pathway was modulated by miR-200b in GC. Western blotting was carried out for exploring the downstream genes of ERBB2/ERBB3 in GC cells after treated with miR-200b mimic, miR-200b inhibitor, or combined with NRG1 siRNA. The results showed that the phosphorylation of ERBB2 or ERBB3 was downregulated in miR-200b mimic GC cells and upregulated in miR-200b inhibitor GC cells. Furthermore, p-ERBB2 or p-ERBB3 expression was decreased by NRG1 siRNA induced by miR-200b inhibitor in GC cells (Figures 5(a) and 5(b)). These findings suggested that miR-200b regulated GC cell invasion and migration via modulating NRG1 through the ERBB2/ERBB3 signaling pathway.

## 4. Discussion

Increasing evidence has displayed that miRs played critical roles in GC metastasis. Therefore, it is very essential to identify the metastasis-related miRs for understanding the potential mechanism in GC development. It has been revealed that miR-200b was involved in the progression of several cancers. For instance, miR-200b took part in bladder cancer migration and invasion as a tumor suppressor [23]. MiR-200b showed an oncogene behavior in colorectal and cervical cancer [24, 25]. Moreover, Tang et al. showed that miR-200b served as a prognostic factor and mediator of GC progression [26]. In this study, the results displayed that miR-200b was dramatically decreased in GC and its downregulation predicted poor prognosis of GC patients. Moreover, we also displayed that miR-200b repressed GC cell invasion and migration.

Tumor metastasis is a complex process involving a series of events [27]. EMT displays an important role in the metastasis of tumors and acts an important biological process in which epidermal malignant cells acquire the ability to migrate and invade. Previous studies have proved the role of miRs in EMT progression [28, 29]. In our study, we displayed that miR-200b was significantly associated with EMT-related markers and reexpression of miR-200b was repressed, whereas silence of miR-200b promoted EMT-associated markers.

NRG1 was reported to act as an oncogene in various tumor development [20, 30]. In this study, we showed that NRG1 was upregulated in GC and it was inversely associated with miR-200b expression. More importantly, NRG1 could reverse the miR-200b effect on GC cell migration and invasion. A previous study displayed that NRG1 exerted its roles in tumors by binding to ERBB2 or ERBB3, resulting in tumor cell proliferation, invasion, and migration [21]. Our study revealed that the phosphorylation of ERBB2 or ERBB3 was markedly inhibited in GC cells after overexpression of miR-200b, while it was elevated after inhibiting miR-200b and decreasing NRG1 overturned the miR-200b inhibitor effect on the ERBB2/ERBB3 signaling pathway.

This study has its limitations that it only investigated the biological functions of the miR-200b/NRG1 axis on cell migration and invasion in vitro. The functional role of miR-200b in in vivo animal experiments is required to explore in the future studies.

In conclusion, miR-200b inhibited GC cell migration and invasion through EMT via the NRG1/ERBB2/ERBB3 signaling pathway, and miR-200b might be a potential biomarker for GC progression, which provides a clue for treating GC.

## Figures and Tables

**Table 1:** Correlation with miR-200b expression and clinicopathological features in gastric cancer patients.

Item	MiR-200b		*p* value
	High (*n* = 29)	Low (*n* = 31)	
Age (years)			0.205
＜60	14	20	
≥60	15	11	

Gender			0.809
Female	15	17	
Male	14	14	

AJCC stage			0.017^*∗*^
I-II	21	13	
III-IV	8	18	

Tumor size, cm			0.009^*∗*^
<3	21	12	
≥3	8	19	

Lymph node metastasis			0.190
Negative	18	14	
Positive	11	17	

Invasion into the serous layer			0.305
Yes	16	13	
No	13	18	

Statistical analyses were performed by the *χ*2 test. ^*∗*^*p* < 0.05 was considered significant.

## Data Availability

The datasets used and/or analyzed during the present study are available from the corresponding author on reasonable request.
